# Sulfhydryl functionalized carbon quantum dots as a turn-off fluorescent probe for sensitive detection of Hg^2+^†

**DOI:** 10.1039/d1ra06527k

**Published:** 2021-11-10

**Authors:** Wei Yao, Yingchen Hua, Zhihong Yan, Chunxian Wu, Feiyan Zhou, Yi Liu

**Affiliations:** College of Pharmacy, Guangdong Pharmaceutical University Guangzhou 510000 China Liuyi915@126.com; School of Pharmaceutical and Chemical Engineering, Guangdong Pharmaceutical University Zhongshan 528400 China; Guangzhou Baiyunshan Weiyi Industrial Co., Ltd Guangzhou 510000 China

## Abstract

Mercury ion (Hg^2+^) is one of the most toxic heavy metal ions and lowering the detection limit of Hg^2+^ is always a challenge in analytical chemistry and environmental analysis. In this work, sulfhydryl functionalized carbon quantum dots (HS-CQDs) were synthesized through a one-pot hydrothermal method. The obtained HS-CQDs were able to detect mercury ions Hg^2+^ rapidly and sensitively through fluorescence quenching, which may be ascribed to the formation of nonfluorescent ground-state complexes and electron transfer reaction between HS-CQDs and Hg^2+^. A modification of the HS-CQD surface by –SH was confirmed using Fourier transform infrared spectroscopy (FTIR) and X-ray photoelectron spectroscopy (XPS). The HS-CQDs sensing system obtained a good linear relationship over a Hg^2+^ concentration ranging from 0.45 μM to 2.1 μM with a detection limit of 12 nM. Delightfully, the sensor has been successfully used to detect Hg^2+^ in real samples with satisfactory results. This means that the sensor has the potential to be used for testing actual samples.

## Abstract 2

Schematic presentation of the synthesis of HS-CQDs and the application as a “turn-off” fluorescent probe for Hg^2+^ detection.

## Graphical abstract

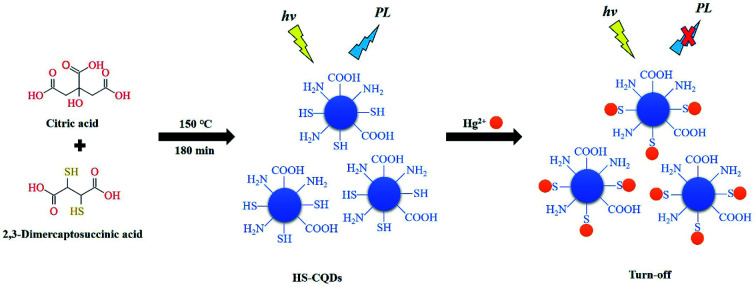

## 1. Introduction

As we all know, with the development of industry and economy, various heavy metal ions have caused relatively serious pollution threatening human health and ecosystems.^1,2^ Mercury ion (Hg^2+^) is one of the most toxic heavy metal ions. It easily accumulates in the human body through the food chain and can cause a series of health problems, such as lung and kidney function damage, chest pain, dyspnea, nervous system damage, and even cancer.^3,4^ Therefore, it is of great significance to establish a highly selective and sensitive detection method for Hg^2+^. There are many traditional methods for the determination of Hg^2+^, such as atomic absorption spectrometry (AAS),^5^ anodic stripping voltammetry (ASV),^6^ inductively coupled plasma atomic emission spectrometry (ICP-AES),^7^ inductively coupled plasma mass spectrometry (ICP-MS),^8^ liquid chromatograph-mass spectrometry (LC-MS)^9^ and other sophisticated methods. However, expensive and complex instruments, a lengthy process and procedures for pre-treatment of complex samples all limit the application of these methods. There have been many novel sensors developed in recent years for measuring Hg^2+^ based on functionalized multi-walled carbon nanotubes,^10^ metal nanoparticles^11,12^ and semiconductor quantum dots (QDs).^13^ Semiconductor QDs have also been widely investigated and used as fluorescent probes due to their unique optical properties and surface modification. Such as, Kumagai *et al.* modified the MOF material on the surface of QDs by dropping metal–organic frameworks (MOFs) precursors into the quantum dot solution and then the ligands were exchanged for pyridine derivatives.^14^ However, general QDs modification methods involve complicated procedures and expensive reagents. But other than that, the requirement of multiple pre-processing steps, harsh synthetic conditions and expensive raw materials all limit the widespread application of these methods.

Recently, carbon quantum dots (CQDs), a new type of fluorescent nanoparticle, has attracted great interest from researchers. CQDs are usually spherical, have a particle size below 10 nm, and emit fluorescence that is strong and stable.^15,16^ Compared with organic dyes, quantum dots and precious metal nanoparticles, CQDs have many advantages, including good water solubility, excellent photostability, great biocompatibility and low toxicity, which make carbon quantum dots can be effectively used in the practical applications of chemical sensors and biosensors.^17,18^ In the above applications, it has been demonstrated surface modification and heteroatomic doping can enhance the efficiency of CQDs.^19,20^ Gui *et al.* combined the red carbon dots (CDs) modified with amino groups on the surface with the blue silicon dots (SiDs) modified with carboxyl groups on the surface by activation coupling of carbondiimide.^21^ According to some researches, these fluorescence sensors are characterized by their sensitivity and selectivity largely due to the affinity of active groups (such as amino and sulfhydryl) on the CQDs surface for the ions they detect.^22–24^

**Scheme 1:**
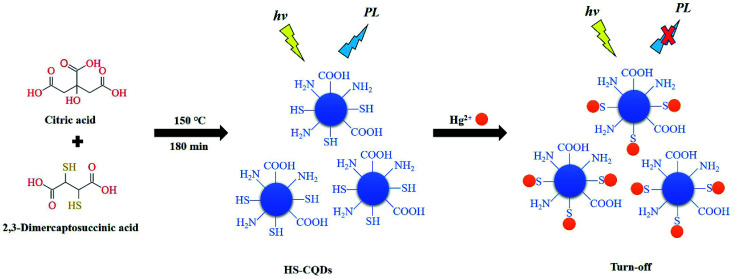
Schematic presentation of the synthesis of HS-CQDs and the application for Hg^2+^ detection.

Hence, because *meso*-2,3-dimercaptosuccinic acid is rich in sulfhydryl groups and able to do react with citric acid at high temperature and pressure, the *meso*-2,3-dimercaptosuccinic acid was used as the sulfur source to synthesize successfully the sulfhydryl functionalized carbon quantum dots (HS-CQDs). This one-pot preparation route is very easy, green and eco-friendly and was successful at introducing sulfhydryl functional groups. Compared with other functional group modification methods, this approach does not require either sophisticated equipment or complex procedures. Because of the chelation between –SH and Hg^2+^,^25^ the prepared HS-CQDs were developed to high selectively detect Hg^2+^ with high accuracy by a novel fluorescence “turn-off” model (Scheme 1). In the presence of ultraviolet light, the prepared HS-CQDs emitted blue fluorescence with high quantum yield (QY) (15.8%), excellent water solubility and stability. In addition, the possible mechanism of detecting Hg^2+^ was also elucidated. The developed Hg^2+^ sensor based on HS-CQDs shows satisfactory detection limit (LOD = 12 nM) and concentration ranges under ideal experimental conditions. To the best of our knowledge, this is lower than most other carbon-based fluorescent probes. What's more important, the results of recovery test showed that the “turn-off” fluorescence reported exhibits high applicability to systems and can be used for the detection of Hg^2+^ in the environment.

## 2. Experimental

### 2.1 Materials and characterization

Citric acid (99.5%) was purchased from Macklin Biochemical Co., Ltd (Shanghai, China). *meso* 2,3-Dimercaptosuccinic acid (98%) and ammonia (25–28%) were purchased from Shanghai Lin En Technology Development Co., Ltd. (Shanghai, China). HgCl_2_ was purchased from Zibo Huatong Chemical Reagent Co., Ltd. (Shandong, China). The metal salts used, including CdCl_2_, ZnSO_4_, KCl, NaCl, BaCl_2_·2H_2_O, CuCl_2_·2H_2_O, MgCl_2_·6H_2_O, NiCl_2_·6H_2_O, MnSO_4_·H_2_O, CaCl_2_, Pb(NO_3_)_2_, FeCl_2_, FeCl_3_·6H_2_O, AgNO_3_ and CrCl_3_·6H_2_O were of analytical purity-grade and used without any further purifications. Ultrapure water was produced with a Ultrapure Water System (Nanjing QuanKun Bio-technology Co., Ltd, China) and used throughout the experiments.

UV-visible absorption (UV-Vis) spectrums were measured by using a A11665 UV-Vis spectrophotometer (Shimadzu, Japan). Fourier transform infrared spectroscopy (FTIR) spectrum were performed on a IRAffinity-1 spectrophotometer (Shimadzu, Japan). The fluorescence measurements were recorded using an LF-1804005 fluorescence spectrophotometer (Thermo Fisher Scientific, China). The slit width was set at 5 nm for both excitation and emission. Transmission electron microscopy (TEM) images were measured by H-7650(HITACHI, Japan). X-ray photoelectron spectroscopy (XPS) were performed on ESCALAB 250 XI (Thermo Fisher Scientific, China). X-ray diffractometer (XRD) pattern was measured by D/max-2200/PC (Rigaku Corporation, Japan).

### 2.2 Synthesis of HS-CQDs

HS-CQDs were synthesized by a one-pot synthetic method. The 1 g citric acid and 0.68 g *meso* 2,3-dimercaptosuccinic acid were placed into a clean, dry beaker containing 25 mL of ultrapure water and 10 mL ammonia was added under rapid stirring to make *meso* 2,3-dimercaptosuccinic acid sufficiently dissolved. Afterward, the mixture was heated at 150 °C for 180 minutes in a Teflon-lined autoclave (40 mL). Cooling until room temperature is reached, the above aqueous solution was bag (1000 Da) for 48 h to remove raw materials. After dialysis, the solution was filtered through 0.22 μm membrane filter to remove the sediment and frozen drying. Finally, the purified HS-CQDs were brown powder and prepared for subsequent use.

### 2.3 Fluorescence QY measurement

The measurement method of fluorescence QY was based on previous studies.^26^ A fraction of the reference substance quinine sulfate (QY = 0.577) was dissolved in 0.05 N sulfuric acid solution as a reference substance, and the emission peak integral area and UV absorbance of the substance to be tested and quinine sulfate were measured at 350 nm (the UV absorbance remained below 0.05). The quantum yield was calculated by the following formula (1):

1 QY_s_ = QY_r_(*F*_s_/*F*_r_)(*A*_r_/*A*_s_)(*η*_s_/*η*_r_)^2^

Specially, QY is the quantum yield, *F* symbolizes the fluorescence emission peak area, *η* is the refractive index of the solvent, and *A* is the absorbance at the excitation wavelength. The subscript “r” refers to standard and “s” refers to the sample.

### 2.4 Detection of Hg^2+^

The sensing system of Hg^2+^ were prepared by mixing 4800 μL of ultrapure water, 100 μL of HS-CQDs (1.0 mg mL^−1^) and 100 μL of Hg^2+^ with different concentrations. Kept the total volume at 4 mL and the final concentration of HS-CQDs was 25 μg mL^−1^. After reacting at room temperature for 1 min, the fluorescence spectra were recorded at the excitation wavelength of 350 nm. The linear regression equation was obtained from fluorescence quench factor (1 − *F*/*F*_0_) *versus* the concentrations of Hg^2+^ data. To test the selectivity for the sensor toward Hg^2+^, different metal ions (Pb^2+^, Cd^2+^, Cr^3+^, Zn^2+^, Cu^2+^, Mn^2+^, Ca^2+^, Mg^2+^, Ba^2+^, Ni^2+^, Fe^2+^, Fe^3+^, Ag^+^, K^+^, Na^+^) with the concentration of 10 μM were added to 25 μg mL^−1^ HS-CQDs solution. For purpose of assessment the anti-interference of the sensor, 10 μM of Hg^2+^ and 13 kinds of metal ions with the concentration of 10 μM were dripped into HS-CQDs solution.

### 2.5 Detection of Hg^2+^ in real sample

In order to investigate the practicality of the sensor, the HS-CQDs sensing system was used for the detection of Hg^2+^ in lake water and waste water samples. These samples were come from the artificial lake and living area of the Zhongshan campus of Guangdong Pharmaceutical University. Then, the lake water and waste water were filtered through a 0.22 μm microporous membrane and centrifuged at 8000 rpm for 10 min to remove the particulate matter. Then 1 mL obtained water samples was added to the mixed solutions with 100 μL of HS-CQDs (1 mg mL^−1^) and 100 μL of different concentrations of Hg^2+^ (0.50 μM, 1.0 μM and 2.0 μM) to obtain fluorescence intensities as the proposed method. Each experiment was done three times in parallel.

## 3. Results and discussion

### 3.1 Characterization of HS-CQDs

**Fig. 1:**
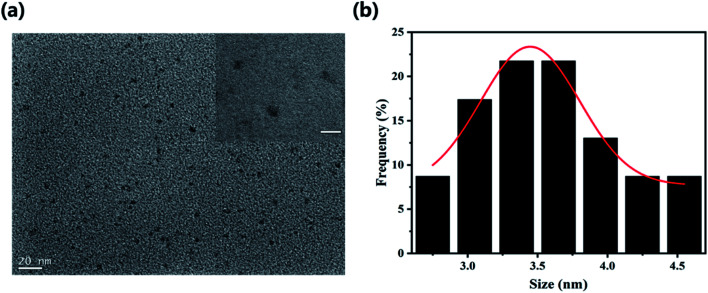
(a) TEM of HS-CQDs (inset: the bar: 10 nm). (b) Size distribution histogram of HS-CQDs.

**Fig. 2:**
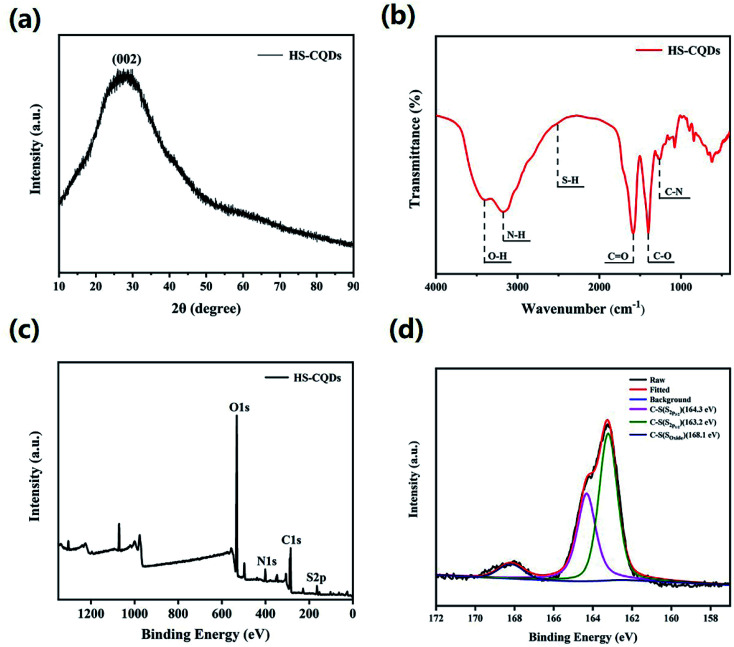
(a) XRD pattern of HS-CQDs. (b) FTIR of HS-CQDs. (c) XPS survey spectrum of HS-CQDs. (d) High resolution XPS spectra of S2p of HS-CQDs.

To explore the surface status and size of HS-CQDs, several characterizations were carried out. As shown in Fig. 1a, the TEM image showed that HS-CQDs were well monodispersed and the morphology of HS-CQDs was spherical. The distributions of particle sizes (Fig. 1b) shows that the HS-CQDs were an average particle size of ∼3.5 nm in the range of 2.75–4.55 nm. The XRD spectrum of the HS-CQDs was shown in Fig. 2a. The sharp peak at around 27° was attributed to the (002) peak of graphite, which means the HS-CQDs have a graphitic structure.^27,28^ FTIR spectrum of HS-CQDs was shown in Fig. 2b and exhibited characteristic peaks of surface functional groups of HS-CQDs. The peak at 3406 cm^−1^ was indicated the stretching vibration of the O–H bond, and the peak at 3175 cm^−1^ was designated as the stretching vibration of the N–H bond of primary amine. The peak of 2510 cm^−1^ was indicated S–H stretching vibration,^29^ suggesting that the sulfhydryl group has been successfully modified to HS-CQDs. The two peaks locating to 1585 cm^−1^ and 1400 cm^−1^ represented the stretching vibration of the C=O and C–O, which was due to the carboxylate in the surface of HS-CQDs. And the peak of 1262 cm^−1^ was attributed to C–N stretching vibrations of amide. The FTIR spectrum demonstrated that the HS-CQDs were successfully functionalized with sulfhydryl, amino and carboxylate.^30,31^

To further explore the functional groups and chemical composition of HS-CQDs, the XPS spectra was investigated. The full scan of the XPS spectrum shown in Fig. 2c showed four different typical peaks at 284.8 eV, 531.6 eV, 401.1 eV and 163.3 eV attributed to carbon (C1s), oxygen (O1s), nitrogen (N1s) and sulfur (S2p) respectively, and their contents were 48.95%, 43.19%, 5.17% and 2.69%, respectively. The high-resolution spectrum of C1s spectrum (Fig. S1a†) was deconvoluted into four peaks at 288.4 eV, 286.5 eV, 284.9 eV and 284.7 eV, which were attributed to C–O–C, C=O, C–N and C–S. The high-resolution spectrum of O1s spectrum (Fig. S1b†) presented two peaks at 532 eV and 531.1 eV, which were owing to C–OH/C–O–C and C=O. The high resolution spectrum of N1s spectrum (Fig. S1c†) was deconvoluted into three peaks at 401.2 eV,400.5 eV and 399.5 eV, corresponding to N–H, N–C and C–N–C, respectively. The high-resolution spectrum of S2p spectrum (Fig. 2d) was divided into three peaks at 168.1 eV, 164.3 eV and 163.2 eV, which derived from the C–S covalent bonds of the thiophene-S for their spin–orbit couplings and sulfate or sulfonate.^24,32^ On the basis of the above results, both FTIR and XPS characterization methods have proved the successful functionalization of HS-CQDs with –COO^−^, –NH_2_ and –SH.

### 3.2 Optical properties

**Fig. 3:**
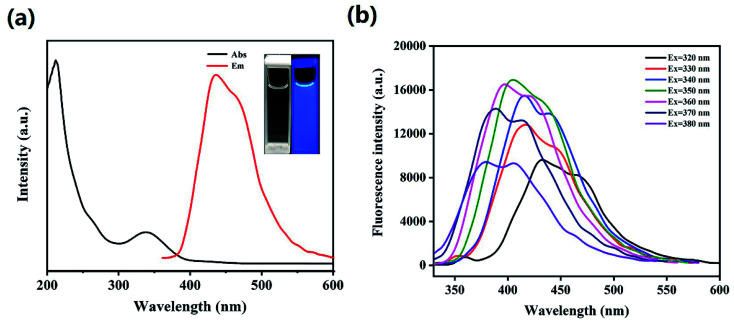
(a) UV-Vis absorption spectrum and fluorescence spectrums(*λ*_ex_ = 350 nm) (inset: photographs of HS-CQDs under the radiation of (left) visible light and (right) 365 nm UV light). (b) Fluorescence spectra of HS-CQDs solutions (25 μg mL^−1^) with different excitation wavelengths from 320 nm to 380 nm.

The HS-CQDs solution was pellucid light yellow in natural light and displayed bluish violet under 365 nm UV light (inset in Fig. 3a). In order to further explore the optical properties of HS-CQDs, HS-CQDs was characterized by UV-vis absorption spectrum and fluorescence spectrum. As shown in Fig. 3a, the weak peak at ∼265 nm was derived from the π–π* transition of C=C.^33^ And there was a strong obviously absorption peak at ∼338 nm attributed to n–π* transition and n–σ* transition, which were relevant to the oxygen-containing functional groups such as C=O and heteroatom groups like –SH, –OH and –NH_2_.^34,35^ The corresponding band gaps of 3.6 eV for HS-CQDs were calculated by using equation *E*_g_ = 1240/*λ*, mainly attributed to the electronic transitions from the valence.^36,37^ As a result of these functional groups, HS-CQDs are more soluble in aqueous solutions and their surface states are changed to enhance their QY. Therefore, with the QY of quinine sulfate as the reference, the QY of HS-CQDs was about 15.8% at the excitation wavelength of 350 nm. This QY of the HS-CQDs prepared in our work is higher than those previous researches reported,^38,39^ which illustrates the extraordinary fluorescent properties and good application prospect of the HS-CQDs in our work.

To inquire into the fluorescent properties of HS-CQDs, the photoluminescence (PL) emission behavior of the HS-CQDs under different excitation wavelengths was investigated. As can be seen from the fluorescence spectrum (Fig. 3b), the fluorescence intensity presented a change law of first increasing and then decreasing with the excitation wavelength increasing from 320 nm to 380 nm. Moreover, the maximum photoluminescence peaks showed a significant shift. This clearly shows the excitation-dependent PL behavior of HS-CQDs, which was correspond to the properties of carbon quantum dots by previous publications.^3,40^ This excitation wavelength-dependent property may be caused by the selection of nanoparticles based on their size (quantum effect) or different emissive traps on the surface of HS-CQDs.^26,41^ When the excitation wavelength is 350 nm, the peak value of fluorescence emission spectrum reaches the maximum. Hence, the wavelength of 350 nm was intended to be the best excitation wavelength.

**Fig. 4:**
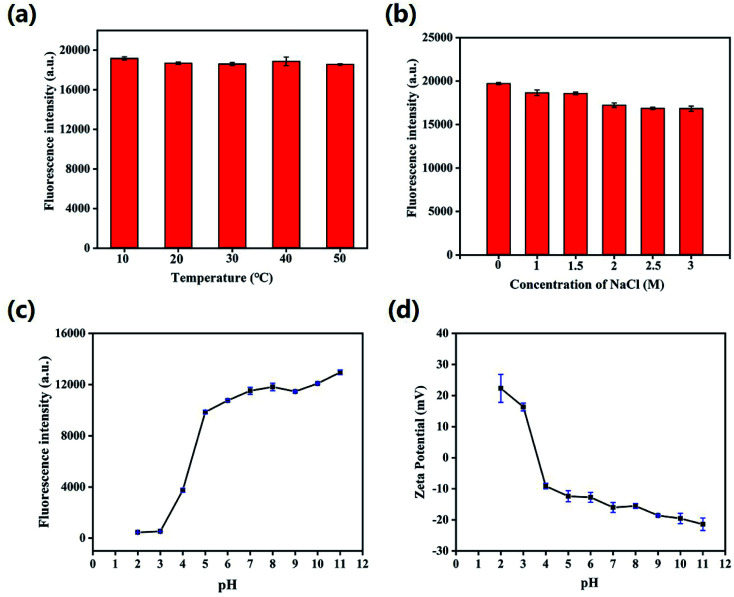
The effect of (a) temperature (10–50 °C), (b) ionic strength in NaCl aqueous solution (1–3 M) and (c) pH (2–11) on the fluorescence intensity of HS-CQDs (25 μg mL^−1^). (d) The effect of pH on the zeta potential of HS-CQDs (25 μg mL^−1^). *λ*_ex_ = 350 nm.

Then, the effects of time, temperature, ionic strength and pH value on the fluorescence stability of HS-CQDs were evaluated. As shown in Fig. S2 and S3,† the color of HS-CQDs solutions hardly changed and still showed a very light yellowish color after being placed for 8 h and 24 h, and the fluorescence of HS-CQDs solutions hardly changed. These results indicated that HS-CQDs has good stability in aqueous solution. Further, it is clear that the fluorescence intensity of HS-CQDs kept stable when the temperature increased from 10 to 50 °C and the concentrations of NaCl aqueous solution ranged from 1 to 3 M, which indicated that temperature and ion strength had almost no effect on the character of HS-CQDs (Fig. 4a and b). As shown in Fig. 4c and d, the fluorescence intensity of HS-CQDs had sharply changed and the zeta potential had changed from positive to negative potentials with the pH changing from 2 to 11, which is due to the extensive protonation–deprotonation of the amide group and of the HS-CQDs.^42^ This suggests that the HS-CQDs is pH responsive, which is similar to other CQDs reported by previous research.^43,44^

### 3.3 Detection of Hg^2+^

In order to obtain optimal reaction conditions, the effect of pH and incubation time on the relationship between fluorescence quenching rate *F*/*F*_0_ and HS-CQDs + Hg^2+^ solution was studied. *F*_0_ and *F* represented PL intensity of HS-CQDs before and after adding Hg^2+^, respectively, and the concentrations of HS-CQDs solution and Hg^2+^ solution were 25 μg mL^−1^ and 10 μM respectively. As shown in Fig. S4,† the PL quenching rate of HS-CQDs has fallen substantially in the pH range of 4–10, and the reaction was complete within 1 minute, which indicated that this fluorescent probe can quickly complete the detection of Hg^2+^ under harsh conditions. Therefore, the best experimental conditions were found when the Hg^2+^ detection was carried out in the pH = 7 ultrapure water after incubating with HS-CQDs for 1 min.

**Fig. 5:**
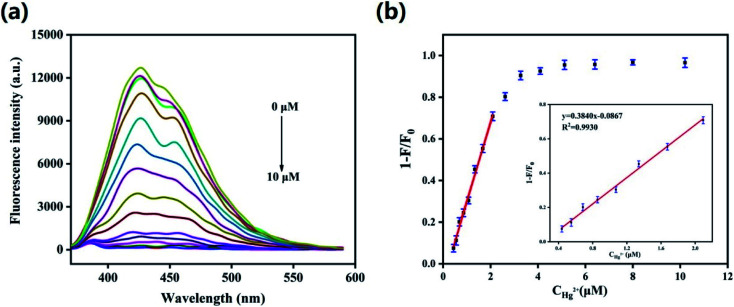
(a) Emission spectra of HS-CQDs (25 μg mL^−1^) with various concentrations of Hg^2+^ in ultrapure water (pH = 7). *λ*_ex_ = 350 nm. (b) Relationship between fluorescence intensity variation and Hg^2+^ concentration ranging from 0 μM to 10 μM. The measurement was carried out in pH = 7 ultrapure water containing 25 μg mL^−1^ HS-CQDs. *λ*_ex_ = 350 nm.

To evaluate the sensitivity of the HS-CQDs for Hg^2+^, the detection limit for Hg^2+^ detection was explored. As shown in the Fig. 5a, the PL intensity was reduced gradually with gradients of Hg^2+^ (0–10 μM) dripped into the HS-CQDs solution (25 μg mL^−1^), implying that the PL intensity of the HS-CQDs is very sensitive to Hg^2+^. The plot of PL quenching rate against concentrations of Hg^2+^ is presented in Fig. 5b. As illustrated in the inset of Fig. 5b, an excellent linear relationship with the concentration of Hg^2+^ in the range of 0.45–2.1 μM is observed. The linear equation was *F*/*F*_0_ − 1 = 0.3824*C*_Hg^2+^_ − 0.08390 with a good linear correlation (the correlation coefficient (*R*^2^) of 0.9943). The limit of detection (LOD) for Hg^2+^ based on a signal-to-noise ratio (S/N) of 3 was approximately 12 nM, which was extremely lower than other fluorescent probes based on carbon materials (Table 1).

**Table 1:** Comparison of the proposed methods with previous methods employed for Hg^2+^ detection based on carbon quantum dots

Methods	Linear range	Detection limit	Ref.
HS-CQDs as“turn-off” fluorescence probe	0.45–2.1 μM	12 nM	This work
Au/N-CQDs as“on–off–on” fluorescence probe	0–41.86 μM	0.118 μM	45
N-CQDs as“off–on” fluorescence probe	0–18 μM	83.5 nM	23
CDs as fluorescence probe	0–80 μM	201 nM	46
N-CQDs as fluorescence probe	0.001–0.1 μM	5.3 nM	47
BN-CDs as fluorescence probe	5–175 μM	2.8 μM	41

**Fig. 6:**
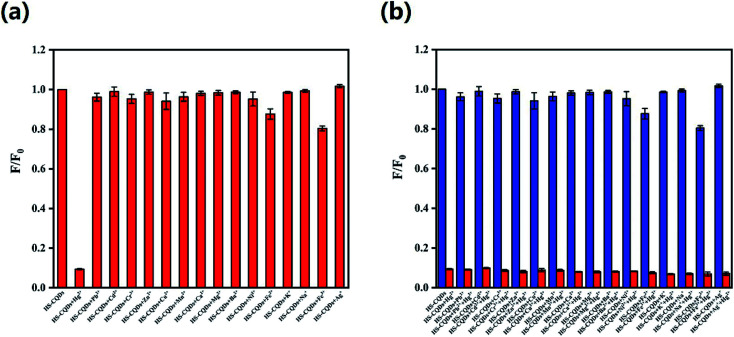
(a) Selectivity and (b) anti-interference of HS-CQDs for the detection to Hg^2+^. The measurement was carried out in pH = 7 ultrapure water containing 25 μg mL^−1^ HS-CQDs or 10 μM metal ions. *λ*_ex_ = 350 nm.

For the sake of the selectivity of the HS-CQDs for Hg^2+^, the PL intensity variations before and after adding 10 μM of metal ions, including Hg^2+^, Cd^2+^, Zn^2+^, K^+^, Na^+^, Cu^2+^, Ni^2+^, Pb^2+^, Fe^2+^, Ba^2+^, Mg^2+^, Mn^2+^, Ca^2+^, Cr^6+^, Fe^3+^, Ag^+^ and Cr^3+^ to the HS-CQDs solution were investigated separately. As shown in Fig. 6a, the PL intensity of HS-CQDs is dramatically decreased (expressed as *F*/*F*_0_) only in the presence of Hg^2+^, and other metal ions caused almost no effect on the fluorescence intensity of the HS-CQDs. Fig. 6b shows the anti-interference performance for HS-CQDs detecting towards Hg^2+^. The blue columns represented the HS-CQDS solution added with other heavy metal ions, and the red columns represented the HS-CQDS solution added with other heavy metal ions and Hg^2+^. It is clearly indicated that there was no significant effect on the PL intensity of the HS-CQDs in the presence of interference metal ions. However, when Hg^2+^ ions were added to the HS-CQDs solutions, remarkable PL quenching was noticed. From these results we can infer the conclusion that the HS-CQDs have an excellent selectivity in detection of Hg^2+^.

### 3.4 Sensing mechanism of HS-CQDs for Hg^2+^

**Fig. 7:**
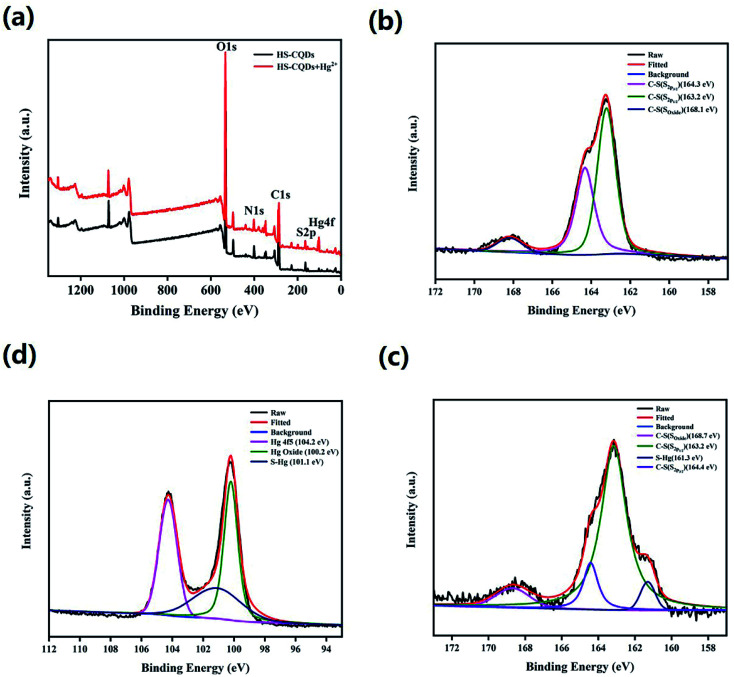
(a) XPS survey spectrum of the HS-CQDs with or without Hg^2+^. (b) High resolution XPS spectra of S2p of HS-CQDs. (c) High resolution XPS spectra of S2p of HS-CQDs–Hg complexes. (d) High resolution XPS spectra of Hg4f of HS-CQDs–Hg complexes.

Zu *et al.* first summarized the various kinds of quenching mechanisms of CQDs.^48^ In general, quenching mechanisms include static quenching, dynamic quenching, Förster resonance energy transfer (FRET),^49^ photoinduced electron transfer (PET),^50^ surface energy transfer (SET),^51^ dexter energy transfer (DET) and inner filter effect (IFE).^52^ In this work, HS-CQDs detect Hg^2+^ through the static quenching effect, which is the nonfluorescent ground-state complex that occurs through the interaction between HS-CQDs and Hg^2+^.^53^ Fig. S5† showed that the absorption peak of HS-CQDs at 338 nm disappeared after dripping Hg^2+^ into the sensing system. This indicated that the fluorescence quenching may due to the chelation between Hg^2+^ and one of the groups of –SH, –COOH and –NH_2_ on HS-CQDs. The full scan of the XPS spectrum of HS-CQDs and HS-CQDs–Hg complexes was shown in Fig. 7a, and the peak at 100.2 eV of Hg4f illustrated the chelation between HS-CQDs and Hg^2+^. The high resolution XPS spectra of S2p of HS-CQDs and HS-CQDs–Hg complexes (Fig. 7b and c), and Hg4f of HS-CQDs–Hg complexes further explained the existence of chelates (Fig. 7d). The peak at 161.3 eV of S2p spectra and the peak at 101.1 eV of Hg4f spectrum were attributed to S–Hg, which demonstrated the chelation between Hg^2+^ and –SH on HS-CQDs.

### 3.5 Application to real sample analysis

In order to evaluate the practicality of this fluorescent probe, the HS-CQDs sensing system was applied to detecting Hg^2+^ in lake water and waste water. As listed in Table 2, the recoveries of Hg^2+^ in real samples are ranging from 97.79% to 105.7%. Further, the relative standard deviations (RSD) of three replicate detections for each sample below 5.0%, and thus, quite acceptable. It indicated that this fluorescent probe used to detect Hg^2+^ in real sample is still sensitive. All these studies have shown that this fluorescent probe is an effective, practical, sensitive and selective fluorescent sensor for monitoring Hg^2+^ levels in real water samples.

**Table 2:** Detection of Hg^2+^ in real samples (*n* = 3)

Sample	Add (μM)	Found (μM)	Recovery (%)	RSD (%)
Lake water	0	0.020	—	1.4
	0.50	0.51	102.6	1.6
	1.0	1.1	105.7	2.0
	2.0	2.0	97.79	0.5
Waste water	0	0.042	—	1.7
	0.50	0.52	104.0	4.1
	1.0	1.1	102.1	3.6
	2.0	2.0	101.7	1.8

## 4. Conclusion

In summary, we first introduced *meso*-2,3-dimercaptosuccinic acid as sulfur source to synthesize HS-CQDs through a simple, low-consumption and pollution-free one-pot hydrothermal method. The size of particles of HS-CQDs on average is 3.5 nm with good quantum yield and fluorescence properties. Based on the above characterization results, it can be seen directly that –SH was successfully modified on the surface of CQDs. In addition, the prepared HS-CQDs were used for Hg^2+^ detection due to their highly selectivity, sensitivity, and effectiveness. Delightedly, it has proved that the fluorescence quenching mechanism of detecting Hg^2+^ is realized by the formation of nonfluorescent ground-state complexes and electron transfer reaction between HS-CQDs and Hg^2+^, leading to the static fluorescence quenching of HS-CQDs. In the fluorescence “turn-off” processes, this fluorescent probe obtained a good linear relationship over Hg^2+^ concentration ranging from 0.45 μM to 2.1 μM with a detection limit of 12 nM. The sensor has been successfully used to detect Hg^2+^ in real samples with satisfactory results. Furthermore, due to the fact that the synthesis method of HS-CQDs was straightforward, rapid, time-saving and cost-effective, it has provided a possibility for the practical application of Hg^2+^ detection with high sensitivity and selectivity in the future.

## Conflicts of interest

There are no conflicts to declare.

## Supplementary Material

RA-011-D1RA06527K-s001
