# Supplementary material for: Sulfhydryl functionalized carbon quantum dots as a turn-off fluorescent probe for sensitive detection of Hg2+
Source: RSC Adv. 2021 Nov 10;11(57):36310–8. doi: 10.1039/d1ra06527k (PMC9043377; doi:10.1039/d1ra06527k)
Supplement: RA-011-D1RA06527K-s001 [file RA-011-D1RA06527K-s001.pdf]

Electronic Supplementary Material (ESI) for RSC Advances.
This journal is © The Royal Society of Chemistry 2021

Sulfhydryl functionalized carbon quantum dots as a turn-off fluorescent probe for sensitive detection of Hg^{2+}

Wei Yao[1] , Yingchen Hua[2] , Zhihong Yan[1], Chunxian Wu[2*], Feiyan Zhou[3*], Yi Liu[1,2,3*]
[1]College of Pharmacy, Guangdong Pharmaceutical University, Guangzhou 510000, China
[2]School of pharmaceutical and chemical engineering, Guangdong Pharmaceutical University, Zhongshan 528400, China
[3]Guangzhou Baiyunshan Weiyi Industrial Co., Ltd, Guangzhou 510000, China
*The first corresponding author: E-maill: Liuyi915@126.com.

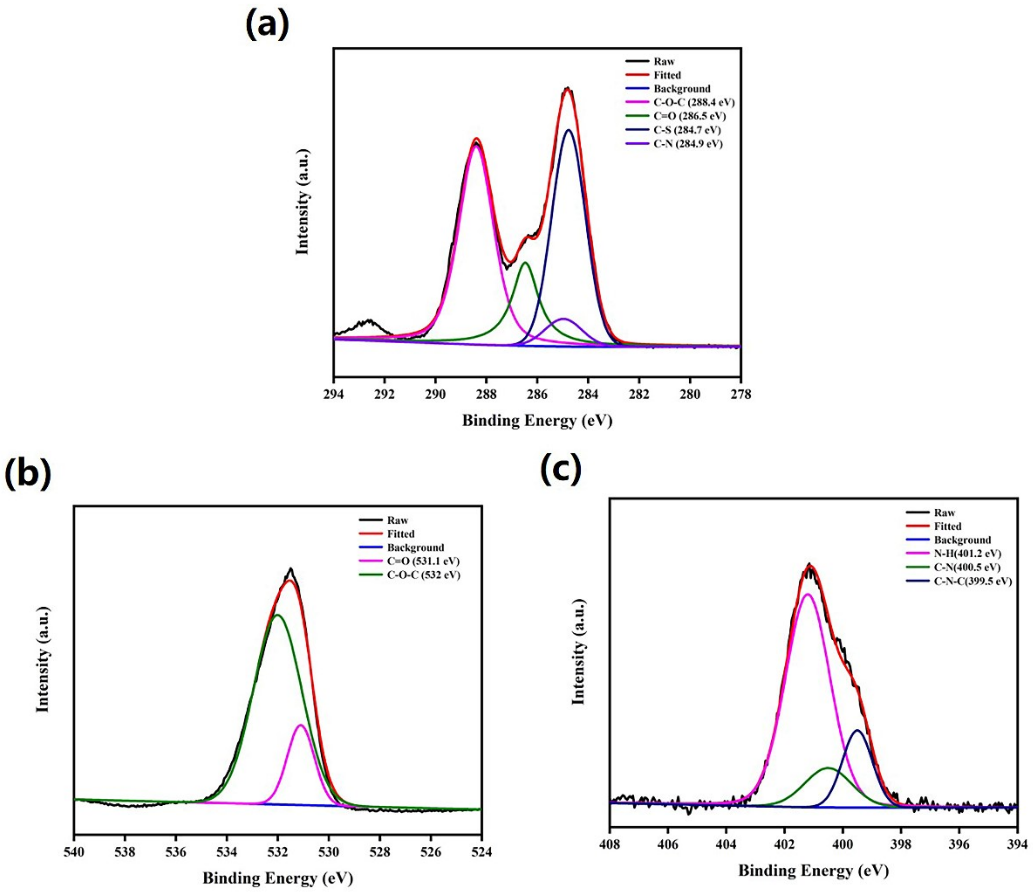

Fig S1. High resolution XPS spectra of **(a)** C1s, **(b)**O1s, **(c)**N1s of HS-CQDs.

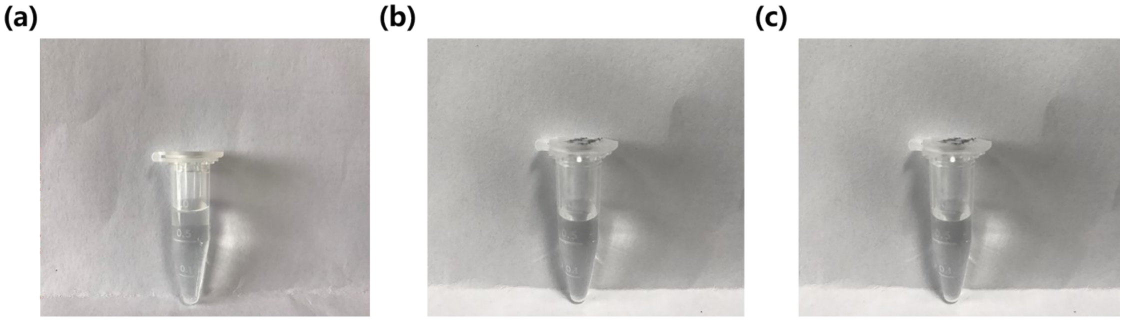

Fig S2. Images of HS-CQDs solutions(1mg/mL) placed for 0h **(a)**, 8h **(b)** and 24h **(c)**.

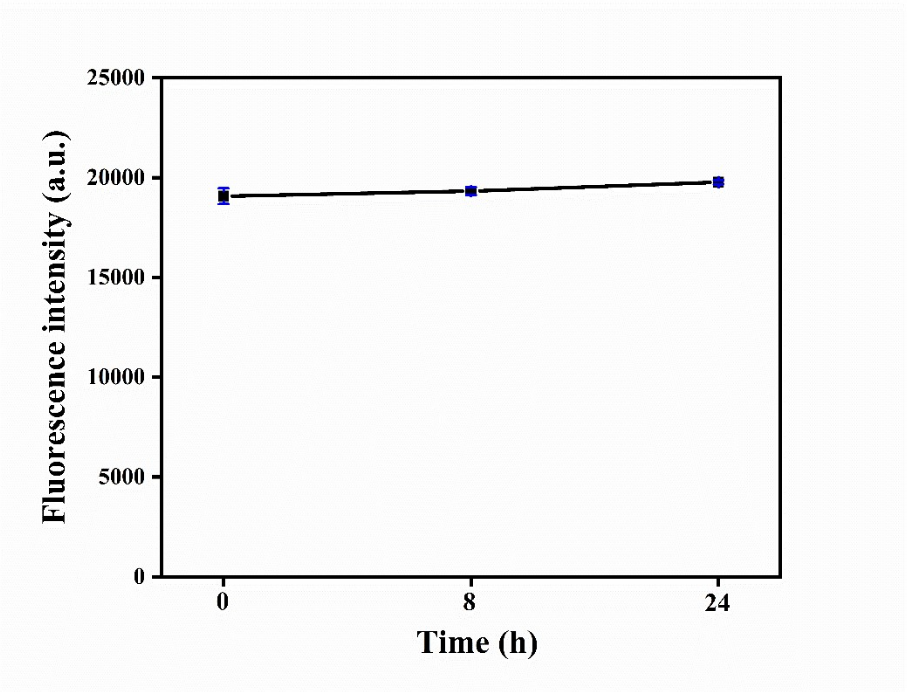

Fig S3. Fluorescence spectra of HS-CQDs solutions (25 μg/mL) placed for 0h, 1h and 2h. λex=350nm.

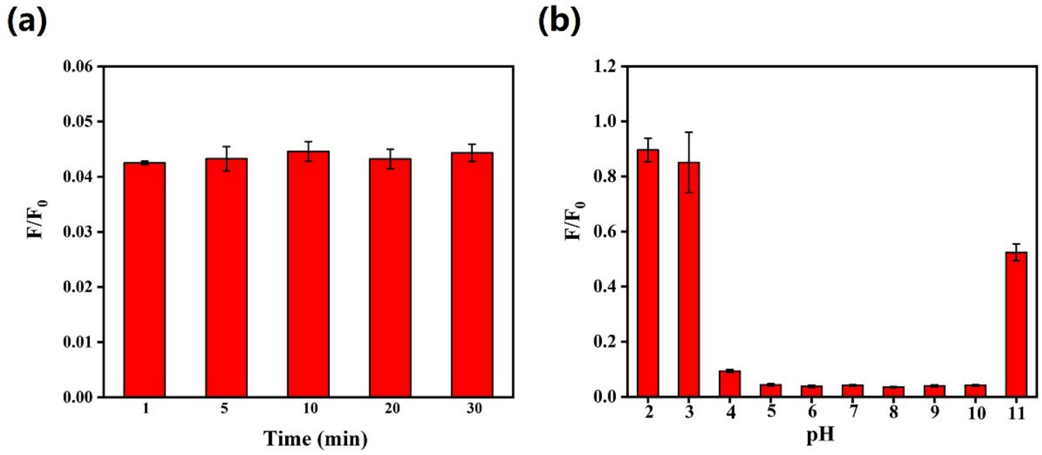

Fig S4. Effect of **(a)** reaction time (1~5min) and **(b)** pH value (2~11) on the detection of Hg^{2+}(10μM) by HS-CQDs(25 μg/mL). λex=350nm.

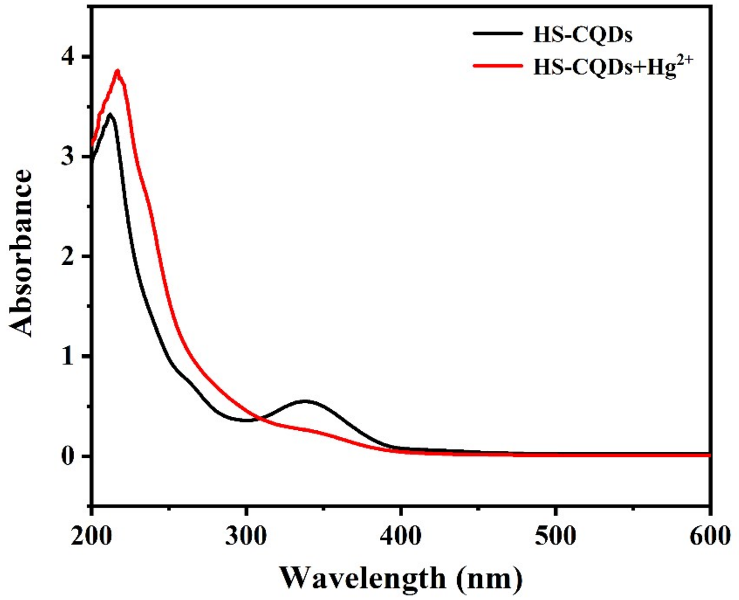

Fig S5. UV-Vis spectra for the HS-CQDs (100 μg/mL) with or without Hg^{2+}(50 μM).
